# Chronic kidney disease exacerbates ischemic limb myopathy in mice via altered mitochondrial energetics

**DOI:** 10.1038/s41598-019-52107-7

**Published:** 2019-10-29

**Authors:** Fabian N. Berru, Sarah E. Gray, Trace Thome, Ravi A. Kumar, Zachary R. Salyers, Madeline Coleman, Dennis Le, Kerri O’Malley, Leonardo F. Ferreira, Scott A. Berceli, Salvatore T. Scali, Terence E. Ryan

**Affiliations:** 1 Department of Applied Physiology and Kinesiology, University of Florida, Gainesville, FL USA; 2 Division of Vascular Surgery and Endovascular Therapy, University of Florida, Gainesville, FL USA; 3 Center for Exercise Science, University of Florida, Gainesville, FL USA; 4 Malcolm Randall Veteran Affairs Medical Center, Gainesville, FL USA

**Keywords:** Chronic kidney disease, Peripheral vascular disease

## Abstract

Chronic kidney disease (CKD) substantially increases the severity of peripheral arterial disease (PAD) symptomology, however, the biological mechanisms remain unclear. The objective herein was to determine the impact of CKD on PAD pathology in mice. C57BL6/J mice were subjected to a diet-induced model of CKD by delivery of adenine for six weeks. CKD was confirmed by measurements of glomerular filtration rate, blood urea nitrogen, and kidney histopathology. Mice with CKD displayed lower muscle force production and greater ischemic lesions in the tibialis anterior muscle (78.1 ± 14.5% vs. 2.5 ± 0.5% in control mice, P < 0.0001, N = 5–10/group) and decreased myofiber size (1661 ± 134 μm^2^ vs. 2221 ± 100 μm^2^ in control mice, P < 0.01, N = 5–10/group). This skeletal myopathy occurred despite normal capillary density (516 ± 59 vs. 466 ± 45 capillaries/20x field of view) and limb perfusion. CKD mice displayed a ~50–65% reduction in muscle mitochondrial respiratory capacity in ischemic muscle, whereas control mice had normal mitochondrial function. Hydrogen peroxide emission was modestly higher in the ischemic muscle of CKD mice, which coincided with decreased oxidant buffering. Exposure of cultured myotubes to CKD serum resulted in myotube atrophy and elevated oxidative stress, which were attenuated by mitochondrial-targeted therapies. Taken together, these findings suggest that mitochondrial impairments caused by CKD contribute to the exacerbation of ischemic pathology.

## Introduction

Peripheral arterial disease (PAD) is one of the three major manifestations of systemic atherosclerosis that affects 8 to 12 million Americans^1^ and is the third leading cause of cardiovascular mortality^2^. PAD is caused by atherosclerotic narrowing or occlusion in the lower extremities which leads to a spectrum of life-altering symptomatology, including claudication, ischemic rest pain, and gangrene requiring limb amputation. Complicating the etiology of PAD, patients typically present with one or more comorbid conditions or risk factors that accelerate disease evolution and are associated with poorer health outcomes. Among these, chronic kidney disease (CKD) has been reported to accelerate the development of atherosclerosis, decrease functional capacity and increase the risk of amputation or death in PAD patients^3–5^. Mortality risk for PAD patients with CKD is 2–4 times higher than PAD patients without CKD^6–10^. CKD also increases the likelihood that patients will present with ischemic ulceration or gangrene which substantially increases the risk of limb amputation^4,10,11^. In addition to increasing risk of mortality and amputation, CKD also increases the failure rate of endovascular/revascularization surgical interventions in PAD^3,12,13^. Complicating the mortality and surgical failure risk, administration of contrast dyes during endovascular/revascularization surgeries has been shown to induce acute kidney injury that increases future risk of developing CKD^14,15^. Despite strong epidemiological data linking CKD to worsened PAD health outcomes, the biological mechanisms are unknown.

Notwithstanding an important clinical focus on reestablishing limb blood flow, a strong predictor of morbidity and mortality in PAD patients is muscle function/exercise capacity^16–22^. In fact, several large clinical studies have demonstrated a strong association between mortality and skeletal muscle function in PAD patients^17,20^. Previous reports have also documented evidence of skeletal muscle myopathies and necrosis in PAD patients^23,24^, although its potential role in clinical disease pathology is largely unaccounted for in the current literature. In fact, a recent report specifically linked muscle mitochondrial content (reported as citrate synthase protein abundance) to PAD mortality^25^. Our previous work uncovered a role of skeletal muscle mitochondria and mitochondrial-derived oxidative stress in the worsening of skeletal myopathy observed in diabetic mice, another strong risk factor for PAD^26^. Coupled with this body of work, emerging pre-clinical studies have demonstrated that therapeutically targeting ischemic skeletal muscle metabolism can improve limb blood flow recovery and reduce/prevent tissue necrosis^27,28^. Herein, we aimed to evaluate the impact of renal dysfunction on the development of skeletal myopathy in mice subjected to femoral artery ligation, a mouse model of PAD.

## Methods

### Critical limb ischemia patient experiments

Gastrocnemius muscle specimens were obtained from ten patients with critical limb ischemia (CLI) undergoing limb amputation. A portion of the muscle was frozen for tissue histology and H&E staining (details described below). Following H&E staining, myofiber cross-sectional area was manually measured using ImageJ on 50–100 transverse fibers per patient. All histological analysis was performed by a blinded investigator. Another portion of the fresh muscle specimen was processed immediately for mitochondrial respiration assessment as described in detail below and in previous studies^29^. This study was approved by the institutional review board at the University of Florida and carried out according to the Declaration of Helsinki. All participants were fully informed about the research and informed consent was obtained.

### Animals

C57BL/6J mice (Stock # 000664) were obtained from The Jackson Laboratory and were used at 8–12 weeks of age. All rodents were housed in a temperature (22 °C) and light controlled (12-hour light/12-hour dark) room and maintained on standard chow prior to CKD induction with free access to food and water. All animal experiments adhered to the Guide for the Care and Use of Laboratory Animals from the Institute for Laboratory Animal Research, National Research Council, Washington, D.C., National Academy Press, 1996, and any updates. All procedures were approved by the Institutional Animal Care and Use Committee of the University of Florida and North Florida/South Georgia VA Hospital.

### Induction of chronic kidney disease (CKD)

We utilized an established adenine-diet model^30–35^ to induce CKD in mice. Mice were assigned to a casein-based chow diet for 7 days, followed by induction of renal tubular injury by supplementing the diet with 0.2% adenine for 7 days. Thereafter, mice received a 0.15% adenine diet to chronically maintain CKD for 5 weeks prior to FAL surgery, and subsequently remained on their respective diets (casein or 0.15% adenine) until sacrifice. Control mice receive casein diet only for the duration of the study.

### Assessment of kidney function

Glomerular filtration rate was measured using FITC-inulin clearance as previously described^36,37^. Briefly, FITC-inulin was dissolved in 0.9% NaCl (5% w/v) and the solution was dialyzed in the dark for 24 h in 0.9% NaCl at room temperature with a 1000-kDa dialysis membrane (Spectrum Laboratories), followed by sterile filtering through a 0.22 μm filter (ThermoFisher). FITC-inulin (2 μl/g body weight) was injected retro-orbitally under isoflurane. Blood was collected in heparin coated capillary tubes via a ~1 mm tail snip at 3, 5, 7, 10, 15, 35, 56, and 75 min following the injection. Blood was centrifuged and plasma was diluted (1:20) and loaded into a 96-well plate along with a FITC-inulin standard curve and fluorescence was detected using a BioTek Synergy II plate reader. GFR was calculated as previously described^36,37^ using a two-phase exponential decay in GraphPad Prism. Blood urea nitrogen was also assessed in plasma obtained via tail snip using a commercial kit (Arbor Assays K024) according to the manufacturer instructions.

### Kidney histology

Kidney histology was assessed by standard light microscopy. Briefly, following thoracotomy, kidneys were carefully dissected and weights were obtained. The left kidney was then placed in OCT compound and frozen in liquid nitrogen-cooled isopentane for cryosectioning. 5-µm-thick longitudinal sections were cut using a cryotome (Leica CM3050) at −20C and collected on slides for staining. Standard hematoxylin and eosin staining was performed and images were collected at 20x magnification using automated image capture/tiling in order to image the entire muscle section using an Evos FL2 Auto microscope (ThermoScientific). Masson’s trichrome staining was performed according to the manufacturer’s instructions (Sigma HT15) and kidney fibrosis was quantified from six 20x images per animal by a blinded investigator via manual thresholding in Image J. Fibrotic area was expressed as a percentage of the total image area.

### Animal model of peripheral artery disease

Femoral artery ligation (FAL)^38^ was performed by anesthetizing mice with intraperitoneal injection of ketamine (90 mg/kg) and xylazine (10 mg/kg) and surgically inducing unilateral hindlimb ischemia by ligation and excision of the femoral artery from its origin just below the inguinal ligament. The inferior epigastric, lateral circumflex, and superficial epigastric artery branches of the femoral artery were left intact, thereby preserving collateral perfusion to the limb. Superficial limb necrosis^38^ was not observed in any of the mice. At seven days post-FAL, mice were anesthetized with ketamine/xylazine for muscle tissue procurement and experiments described below. Serum samples were withdrawn via cardiac puncture and mice were euthanized by thoracotomy.

### Laser doppler limb perfusion measurements

Limb perfusion was measured using a laser Doppler flowmeter (moorVMS-LDF, Moor Instruments) prior to surgery, immediately post-surgery, and just prior to sacrifice (day seven post-surgery) under ketamine/xylazine anesthesia. Briefly, the hindlimbs were shaved to remove hair and the laser Doppler probe was carefully placed against the skin of the tibialis anterior and the bottom of the paw. Data were collected continuously for 60 seconds and the average perfusion was calculated. Perfusion recovery in the ischemic limbs was calculated as a percentage of the non-ischemic control limb as previously described^26,28^.

### Skeletal muscle morphology and ischemic lesion area

Skeletal muscle morphology was assessed by standard light microscopy. 10-µm-thick transverse sections from tibialis anterior (TA) were cut using a cryotome and collected on slides for staining. For morphological analyses, standard methods for hematoxylin and eosin (H&E) histological staining were performed and images were obtained at 20x magnification using automated image capture/tiling in order to image the entire muscle section using an Evos FL2 Auto microscope (ThermoScientific). All image analysis was conducted by a blinded investigator using Image J. Ischemic lesions were quantified by manually tracing the area containing necrotic (lysed muscle cell membranes) and regenerating (fibers with centralized nuclei) myofibers and the injured area was expressed as a percentage of the total muscle area. Regenerating myofibers were quantified by manual counting of fibers with centralized nuclei in six randomly selected 20x images per muscle section. Non-myofiber area was quantified by thresholding images to obtain the pixel area of tissue between myofibers.

### Immunofluorescence microscopy

Skeletal myofiber cross-sectional area (CSA) and vessel density were assessed by IF microscopy. 10-µm-thick transverse sections were cut from tibialis anterior (TA) muscle frozen in liquid nitrogen cooled isopentane in optimum mounting medium (OCT) using a Leica 3050S cryotome. Muscle sections were fixed with 4% paraformaldehyde (in PBS) for five minutes at room temperature followed by ten minutes of permeabilization with 0.25% triton X-100 in PBS. Sections were blocked for 1 hr at room temperature with PBS supplemented with 5% goat serum and 1% BSA, and incubated overnight at 4 C with a primary antibody for laminin (Sigma L9393, 1:100) to label the myofibers membrane. Following four washes with PBS, muscle sections were incubated with Alexa-Fluor secondary antibodies (ThermoScientific, 1:250) as well as Dylight594 conjugated Griffonia simplicifolia I isolectin B4 (Vector Laboratories, DL-1207) to label endothelial cells (i.e. capillaries). Coverslips were mounted with Vectashield hardmount containing DAPI (Vector Laboratories, H-1500). Images were obtained at 20x magnification using an Evos FL2 Auto microscope (ThermoScientific). All image analysis was conducted by a blinded investigator.

### Assessment of muscle contractile function

Immediately following sacrifice, both limbs were amputated and immediately transferred to a dish containing ice cold bicarbonate-buffered solution (137 mM NaCl, 5 mM KCl, 1 mM MgSO_4_, 1 mM NaH_2_PO_4_, 24 mM NaHCO_3_, and 2 mM CaCl_2_) equilibrated with 95% O_2_ - 5% CO_2_ to maintain pH ∼7.4. The soleus from each leg was quickly excised under a stereo-zoom microscope. We used 4-0 silk suture to tie the proximal tendon to a Dual-Mode Muscle Lever System (300C-LR; Aurora Scientific, Aurora, ON, Canada). The distal tendon was attached to a secured glass rod using a loop of suture. We mounted the muscle between two platinum electrodes submerged in a water-jacketed organ bath containing bicarbonate-buffered solution at room temperature and continuously gassed with 95% O2 - 5% CO2. We adjusted the bundle length to attain maximal twitch tension (optimal length, L_0_), increased the temperature of the organ bath to 37 °C, and allowed 5 min for thermos-equilibration. We then measured isometric forces at stimulation frequencies of 1, 30, 50, and 200 Hz using a biphasic high-power stimulator (701C, Aurora Scientific) delivered with current of 600 mA, pulse duration 0.25 ms, train duration 500 ms. Each stimulation train was interspersed by 1 min intervals. The muscle stimulation and data collection were controlled through an automated software (DMC, Aurora Scientific). Isometric forces were normalized by cross sectional area, which was estimated by measuring muscle weight and length at L_O_. Muscle weight was divided by length multiplied by 1.06 g/cm^3^, the density of mammalian skeletal muscle^39^.

### Preparation of permeabilized muscle fibers

A portion of the red gastrocnemius muscle was dissected and immediately placed in ice-cold buffer X (50 mM K-MES, 7.23 mM K_2_EGTA, 2.77 mM CaK_2_EGTA, 20 mM imidazole, 20 mM taurine, 5.7 mM ATP, 14.3 mM phosphocreatine, and 6.56 mM MgCl_2_-6H_2_O, pH 7.1) for preparation of permeabilized fiber bundles as previously described^40,41^. Fiber bundles were separated along their longitudinal axis using needle-tipped forceps under magnification (MX6 Stereoscope, Leica Microsystems, Buffalo Grove, IL, USA), permeabilized with saponin (30 μg/ml) for 30 minutes at 4 °C on a nutating mixer, and subsequently washed in cold buffer Z (105 mM K-MES, 30 mM KCl, 1 mM EGTA, 10 mM K_2_HPO_4_, 5 mM MgCl_2_-6H_2_O, 0.5 mg/ml BSA, pH 7.1) for 15 minutes until analysis. At the conclusion of each experiment, PmFBs were washed in double-distilled H_2_O to remove salts, freeze-dried (Labconco), and weighed. Myofiber bundle sizes were 0.2–0.6 mg dry weight.

### Mitochondrial respiration measurements

High-resolution O_2_ consumption measurements^42^ were conducted at 37 °C in buffer Z (in mmol/l) (105 K-MES, 30 KCl, 1 EGTA, 10 K_2_HPO_4_, 5 MgCl_2_6H_2_O, 0.5 mg/ml BSA, pH 7.1), supplemented with creatine monohydrate (20 mM), using the OROBOROS O2K Oxygraph. A substrate inhibitor titration protocol was performed as follows: 2 mmol/l Malate + 10 mmol/l Glutamate (State 2 respiration), followed by the addition of 4 mmol/l ADP to initiate State 3 respiration supported by Complex I substrates, convergent electron flow through complexes I and II was initiated with the addition of 10 mmol/l Succinate, 10 μmol/l Rotenone was subsequently added to inhibit Complex I, followed by 10 μmol/l Cytochrome C to test the integrity of the mitochondrial membrane, Complex IV supported respiration was examined using the electron donor N,N,N’,N’-tetramethyl-p-phenylenediamine (TMPD) at 0.4 mmol/l in the presence of 2 mmol/l Ascorbate (to limit auto-oxidation of TMPD) and 5 μmol/l of Antimycin A (to prevent reverse electron flow through Complex III). The rate of respiration was expressed as pmol/sec/mg fiber dry weight. All respiration measurements were conducted at 37 °C and a working range [O2] of ~350 to 200 μM.

### Mitochondrial H_2_O_2_ Emission

Mitochondrial H_2_O_2_ emission was measured in permeabilized myofiber bundles fluorometrically at 37 °C via the Amplex Ultra Red (10 μM)/horseradish peroxidase (HRP: 3 U/mL) detection system (Ex:Em 565:600, using a Horiba Fluorolog) as previously described^42^. Experiments were initiated by first collecting an eight-minute background measurement with the fiber bundle added to assay buffer (in mmol/l) (105 K-MES, 30 KCl, 1 EGTA, 10 K_2_HPO_4_, 5 MgCl_2_6H_2_O, 0.5 mg/ml BSA, pH 7.1, with addition of 10 μM Amplex Ultra Red, 3 U/mL HRP, and 20 U/mL superoxide dismutase), followed by sequential additions of 10 mM succinate, and 1 μM auranofin to inhibit matrix H_2_O_2_ buffering. Fluorescence units were converted to pmols of H_2_O_2_ using a standard curve.

### Citrate synthase activity

Citrate synthase activity was assessed in snap frozen human gastrocnemius muscle specimens obtained from patients as described above. Briefly, ~30 mg of muscle was homogenized by hand in Cellytic M (Millipore-Sigma). Following homogenization, lysates were centrifuged for 15 minutes at 4000xG at 4 °C. The supernatant was collected and protein quantification was performed using a bicinchoninic acid (BCA) assay (ThermoScientific). Citrate synthase activity was performed using a colorimetric enzyme assay kit from Millipore-Sigma (CS0720) according the manufacturer instructions.

### Muscle cell culture

C2C12 muscle cells were obtained from ATCC (CRL-1772) and cultured in DMEM supplemented with 10% FBS and 1% penicillin/streptomycin, in standard conditions (37 °C, 5% CO_2_). Myoblast differentiation will be initiated by serum withdrawal using DMEM supplemented with 2% heat-inactivated horse serum and 1% penicillin/streptomycin.

### Myotube atrophy

Differentiated myotubes were exposed to 5% serum obtained from either control or CKD mice for 24 hours. After 24 h exposure to mouse serum, cells were washed with PBS, fixed with 100% methanol for 10 min, left to air dry for 10 min, and incubated with primary antibody against sarcomeric myosin (MF 20 was deposited to the DSHB by Fischman, D.A. (DSHB Hybridoma Product MF 20)) at 1:25 in blocking solution for one hour at 37 °C. Cells were then washed 3x in PBS, followed by incubation with 1:250 secondary antibody (AlexaFluor594, mouse IgG2b, ThermoFisher) for one hour at 37 °C. Some cell plates were placed in Hank’s balanced salt solution and put into a cakepan hypoxia chamber which was flushed with nitrogen gas for ~10 min, sealed and placed into the culture incubator at 37 C for 4 hours to mimic the limb environment in PAD. Following the 4-hour hypoxia and nutrient deprivation (HND) cells were fixed with methanol and stained as described above. Cells were washed three times in PBS, then imaged using automated capture routines on an Evos FL Auto 2 inverted fluorescent microscope (ThermoFisher). Sixty-four 20x images were captured per well (N = 4 wells per group) and analyzed using custom written routines in CellProfiler (Broad Institute) to assess MF20+ area (myotube area). All processing procedures were performed uniformly over the entire set of images using batch processing modes to remove any human input. Additional experiments were performed in the CKD group in the presence of cyclosporine A (1 μM, a mitochondrial permeability transition pore inhibitor) or mitoTEMPO (5 μM, a mitochondrial targeted antioxidant).

### Myotube ROS production

After 24 hours of treatment with 5% mouse serum, myotubes were washed twice with HBSS and incubated with 500 nM MitoSox (ThermoFisher) for 15 min in HBSS prior to imaging. Sixteen 20x images were captured per well (N = 4–5 wells per group) and analyzed using custom written routines in CellProfiler (Broad Institute) to assess MitoSox mean intensity. All processing procedures were performed uniformly over the entire set of images using batch processing modes to remove any human input.

### Statistical analysis

Data are presented as mean ± SEM or SD as indicated in figure legends. Comparisons between 2 groups were performed by Student’s unpaired two-tailed *t*-test. Comparisons of data with more than 2 groups were performed using two-way ANOVA with Tukey’s post-hoc multiple comparisons. Repeated-measures ANOVA was performed when appropriate. All statistical analysis was performed in GraphPad Prism (Version 6.0). In all cases, *P* < 0.05 was considered statistically significant.

## Results

### CLI patients with CKD display worsened ischemic myopathy

**Figure 1:**
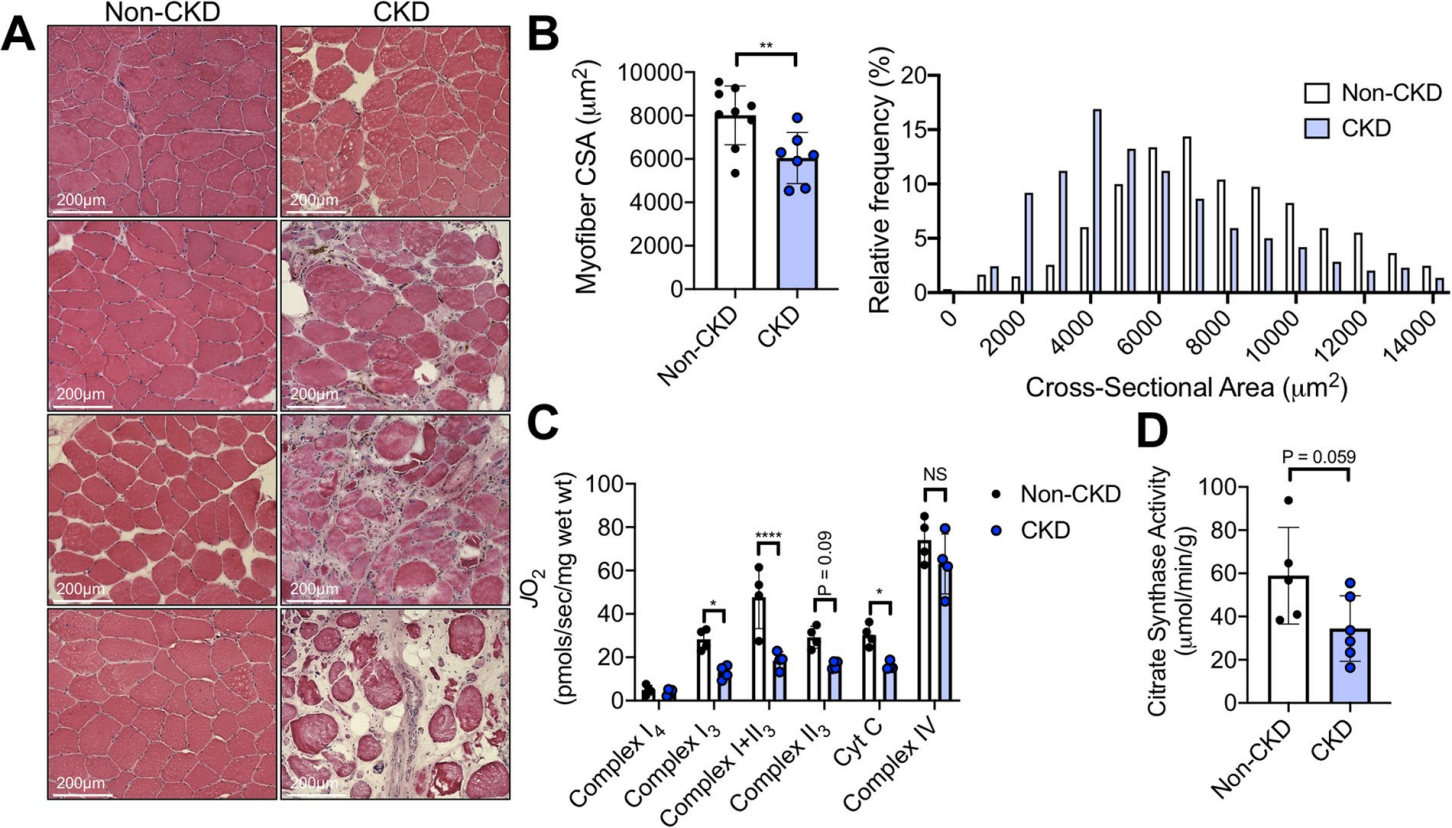
CLI patients with CKD display more severe ischemic skeletal myopathy. Gastrocnemius muscle specimens were obtained from CLI patients (N = 9 non-CKD, N = 7 CKD) undergoing limb amputation. (**A**) Representative H&E images from eight patients. (**B**) Quantification of mean myofiber cross sectional area as well as a histogram displaying the relative frequency of myofiber areas by group. (C) Myofiber mitochondrial respiratory capacity was assessed using sequential additions of glutamate malate (Complex I_4_), ADP (Complex I_3_), succinate (Complex I + II_3_), cytochrome c (to test quality of myofiber preparations), and TMPD/ascorbate (Complex IV). (D) Citrate synthase activity was measured in gastrocnemius muscle lysates from patients. *P < 0.05,*P < 0.01, ****P < 0.0001 using unpaired, two-tailed *t*-test. Error bars represent SEM.

The impetus for this project stems from the clinical observation that PAD patients with CKD present with more severe symptoms placing them at greater risk for limb amputation or death when compared with patients that have normal renal function^5,6,8,10,43,44^. To begin to explore the impact of CKD on ischemic skeletal myopathy, we obtained muscle specimens from the gastrocnemius muscle of CLI patients undergoing limb amputation as result of severe ischemic rest pain and/or non-healing ulcers/gangrene of the lower foot. CLI patients without CKD (N = 9, Age = 68.5 ± 4.9 years, 1 female, two current smokers) all displayed eGFR greater than 60 ml/min/1.73 m^2^ with serum creatinine of 0.66 ± 0.24 mg/dL. The majority of CLI patients (8 out of 10) had non-compressible vessels which prohibited accurate ankle-brachial index measurements. CLI patients with CKD (N = 7, Age = 66.8 ± 4.6 years, all males, eGFR = 32.3 ± 7.2 ml/min/1.73 m^2^, creatinine = 1.98 ± 0.38 mg/dL, one current smoker) displayed a worsened histological myopathy, manifested by myofiber atrophy (Fig. 1A,B), the appearance of interstitial inflammation, small/irregular shaped myofibers, myofibers with centralized nuclei, and necrotic myofibers. CLI patients with CKD also exhibited lower rates of mitochondrial oxygen consumption in permeabilized myofibers bundles (Fig. 1C) and a trend towards lower citrate synthase activity (index of mitochondrial content) was also observed (Fig. 1D).

### CKD mice have normal muscle perfusion recovery and capillary density

**Figure 2:**
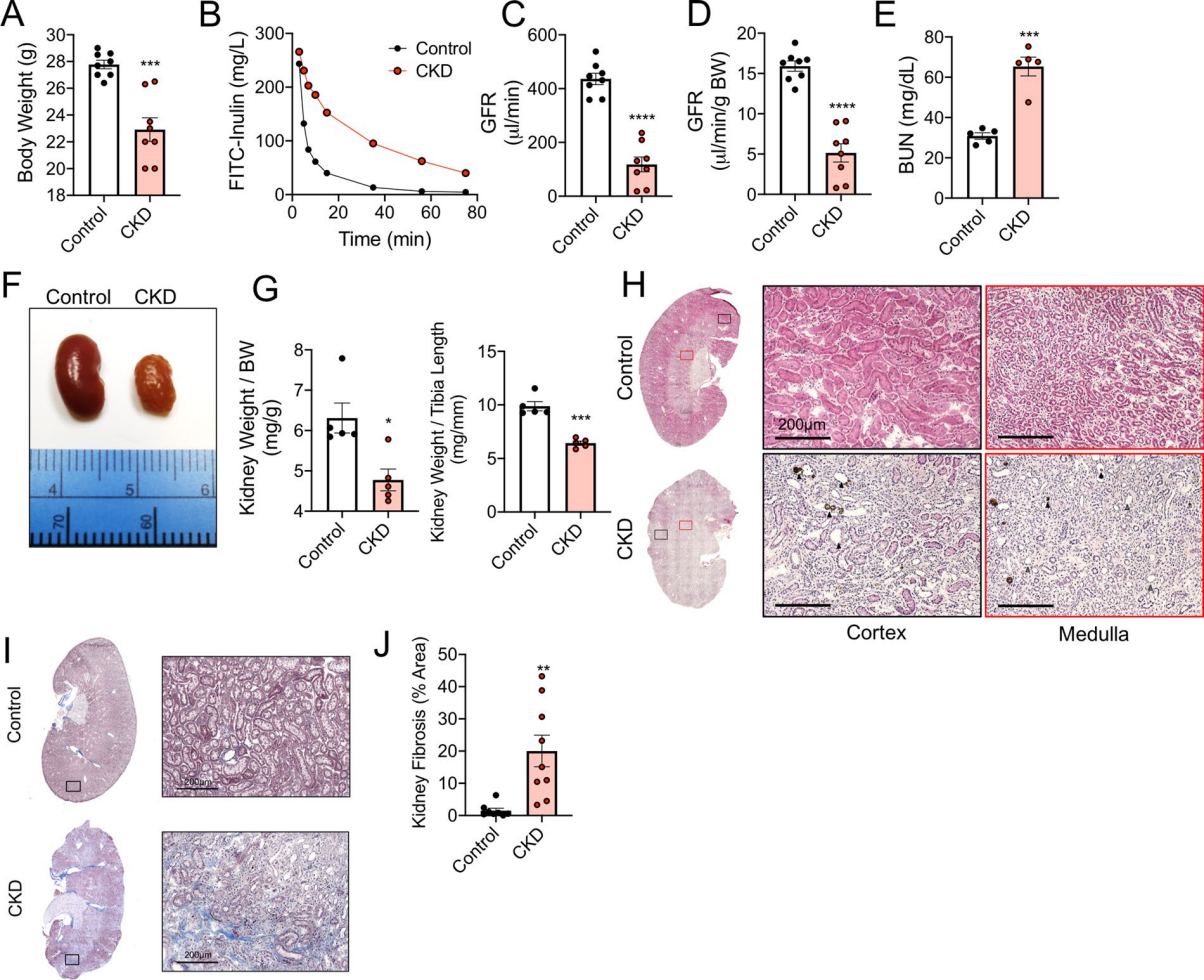
Validation of CKD mouse model. Adult C57BL/6J male mice were fed either a casein control diet (Control) or adenine supplemented (CKD) diet for six weeks. (**A**) CKD mice exhibited lower body weight. CKD was confirmed by measurements of glomerular filtration rate (GFR). (**B**) Representative FITC-Inulin decay curves for control and CKD mice. (**C**) Quantification of GFR and (**D**) GFR normalized to total body weight (N = 8/group). (**E**) Blood urea nitrogen (BUN) was also elevated in CKD mice (N = 5/group). Kidneys from CKD mice also displayed abnormal gross anatomy (**F**), lower kidney weights (**G**). Representative H&E (20x) images of left kidneys (sectioned longitudinally) from control and CKD mice indicate of renal pathology including loss of tubular brush border with interstitial inflammation (grey arrow heads) as well as tubular dilation and crystal formation (black arrow heads). (**I**) Representative images of kidney sections stained with Masson’s Trichrome and (**J**) quantification of kidney fibrosis (blue stained areas). *P < 0.05, **P < 0.01, ***P < 0.001, ****P < 0.0001 vs. control using unpaired, two-tailed *t*-test. Error bars represent SEM.

To examine this clinical observation, we utilized an established rodent CKD model via dietary adenine^30,32–34,45–49^. Mice that received the adenine diet displayed lower body weight (Fig. 2A), and CKD was confirmed by a ~70% reduction in glomerular filtration rate (GFR) compared with casein-fed control mice (Fig. 2B–D). Blood urea nitrogen (BUN) was also significantly higher in CKD mice (30.8 ± 1.6 vs. 65.3 ± 4.6 in CKD, *P* = 0.0001) (Fig. 2E). Kidneys from CKD mice appeared pale and granular, while kidney size was also significantly smaller compared with control mice (Fig. 2F,G). Histological analyses of the kidney demonstrate that CKD mice exhibit tubular luminal dilation with crystals, as well as marked interstitial inflammation (Fig. 2H). Consistent with human CKD kidney pathology, Masson’s trichrome staining revealed significant fibrosis in CKD kidneys with a calculated percentage of fibrotic area of 20.0 ± 14.7% in CKD versus 1.5 ± 2.1% in control mouse kidneys (*P* = 0.0031, N = 8–9/group).

**Figure 3:**
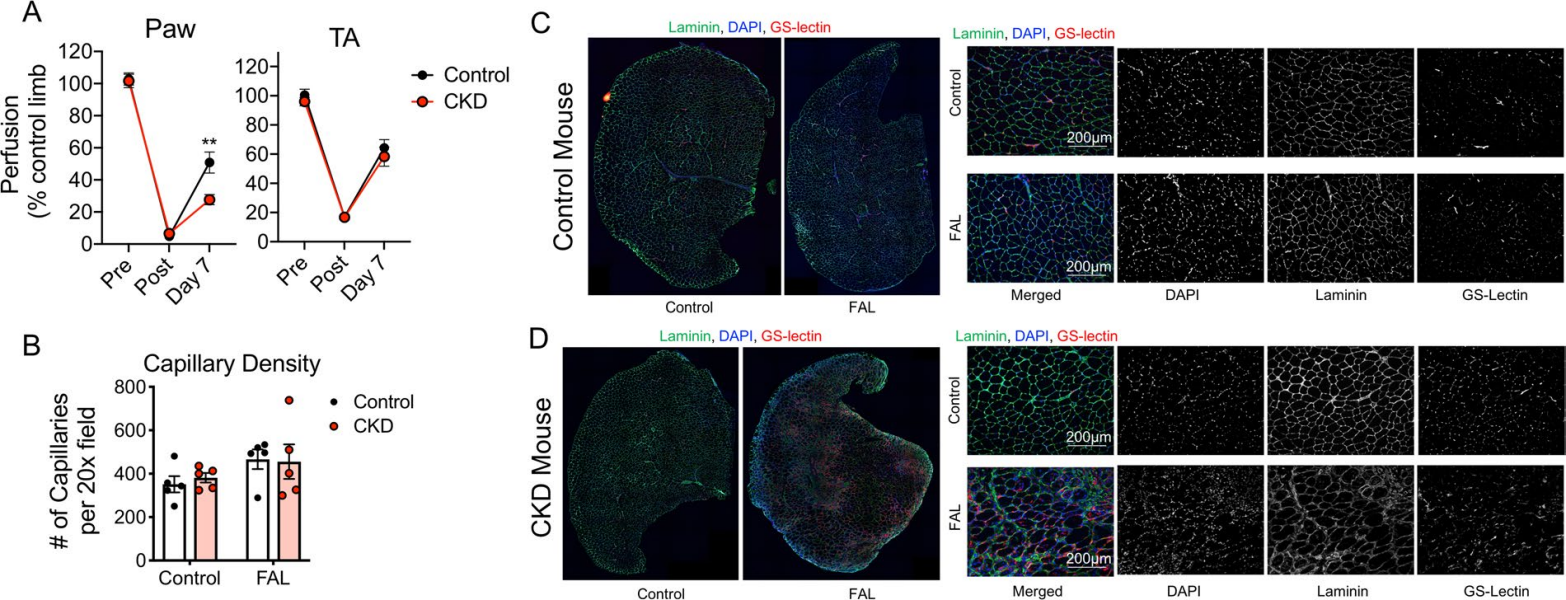
CKD exhibit normal perfusion recovery and capillary density. (**A**) Laser Doppler flowmetry measurements of the paw and TA muscles (normalized as a percentage of the contralateral control limb) demonstrated normal perfusion recovery in CKD mice. (**D**) Total capillary density (labeled by Dylight594-conjugated GS-lectin) was not different between control and CKD mice in either limb muscle. (**C**,**D**) Representative IF images of the TA muscle from control and CKD mice labeled with laminin (myofibers), DAPI (nuclei), and GS-lectin (capillaries). N = 5/group for capillary measurements, N = 9/group for perfusion measurements. **P < 0.01 using two-tailed *t*-test. Error bars represent SEM. FAL = femoral artery ligation.

Limb perfusion was assessed using laser Doppler flowmetry prior to surgery, immediately after surgery, and at sacrifice (day 7 post-surgery). Interestingly, CKD mice displayed normal perfusion recovery in the tibialis anterior muscle but a slight decrease in perfusion recovery of the distal paw (Fig. 3A). Total capillaries were also labeled on sectioned TA muscles using Dylight-594 conjugated lectin. Moreover, total capillary density was not different between control and CKD mice in either limb (Fig. 3B–D). In fact, both control and CKD mice displayed a modest increase in capillary density in the ischemic limb muscle, consistent with an adaptive increase in angiogenesis that results from ischemia.

### CKD mice display greater ischemic muscle myopathy

**Figure 4:**
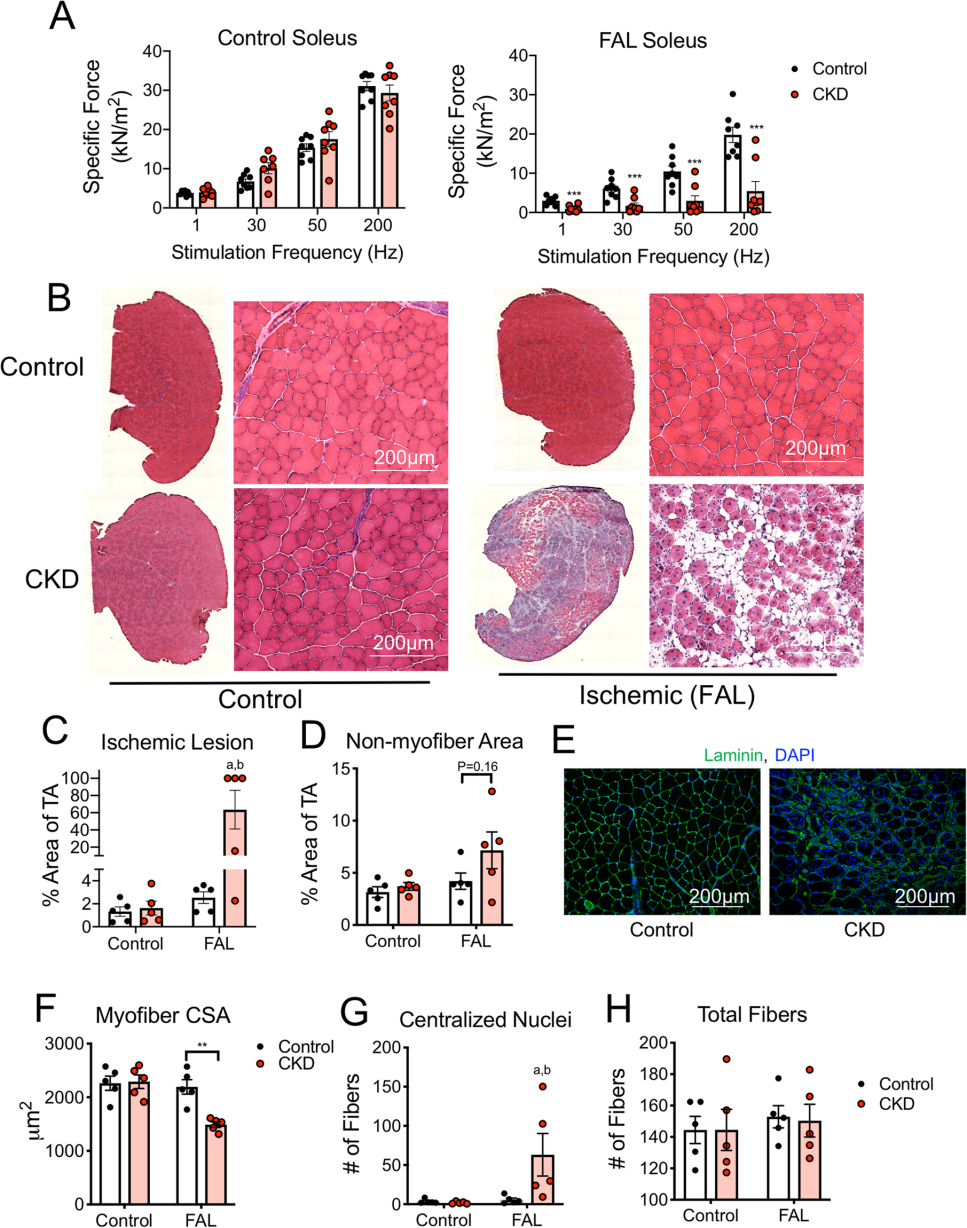
CKD mice display greater ischemic muscle myopathy. C57BL/6J mice were given either a control or adenine-supplemented diet to induce CKD, followed by femoral artery ligation. (**A**) Soleus muscle contractile function (specific force) was assessed in both control and FAL limbs. (**B**) Representative H&E images of the entire TA muscle and 20x magnification. (**C**) CKD mice displayed greater ischemic lesions (amount of TA muscle injured) and (**D**) more non-muscle area within the TA. (**E**) Representative IF images from FAL TA muscles of control and CKD mice stained for laminin and DAPI to label myofibers membranes and nuclei. (**F**) Mean myofibers cross-sectional area (CSA) was measured in both control and FAL limbs. (**G**) CKD mice also displayed a greater number of myofibers with centralized nuclei, however, (**H**) total myofibers were not different between groups. **P < 0.01, ***P < 0.001 using two-tailed unpaired *t*-test. ^a^*P* < 0.05 vs. non-ischemic control limb (within group), ^b^*P* < 0.05 vs. control mice (between group) using ANOVA. N = 5–9/group. Error bars represent SEM. FAL = femoral artery ligation.

Next, we sought to examine the impact of CKD on ischemic muscle health and function. To this end, soleus muscle contractile function was assessed at seven days post-FAL. Specific force (absolute force normalized to muscle cross-sectional area) production at both submaximal and maximal stimulation frequencies was significantly lower in the ischemic muscle of CKD mice when compared with ischemic muscles of control mice (Fig. 4A). Interestingly, CKD mice did not exhibit significant impairments in muscle force in the non-ischemic control muscles. Mice with CKD displayed marked muscle injury, indicated by greater ischemic lesion area (2.5 ± 1.1% vs. 63.5 ± 50.1% in CKD, *P* = 0.026) and a non-significant increase (~50%) in non-myofiber area (*P* = 0.16) (Fig. 4B–D). Myofiber membranes were also labeled with antibodies for laminin to measure myofiber cross-sectional area (CSA). CKD mice displayed a significant reduction in myofiber CSA in the ischemic limb (2194 ± 135 μm^2^ vs. 1489 ± 52 μm^2^ for ischemic limbs in control and CKD mice respectively, *P* = 0.0012) (Fig. 4E). Consistent with a greater level of muscle injury, CKD mice had a significantly greater number of regenerating myofibers (Fig. 4F). The exacerbation of ischemic muscle injury in CKD mice is striking considering that these experiments were performed in C57BL/6J mice, which are known to be resistant to ischemic injury^28,38,50–55^, implying that CKD overrides the genetic protection from ischemic injury in these mice.

### CKD mice suffer from a severe ischemic mitochondriopathy in skeletal muscle

**Figure 5:**
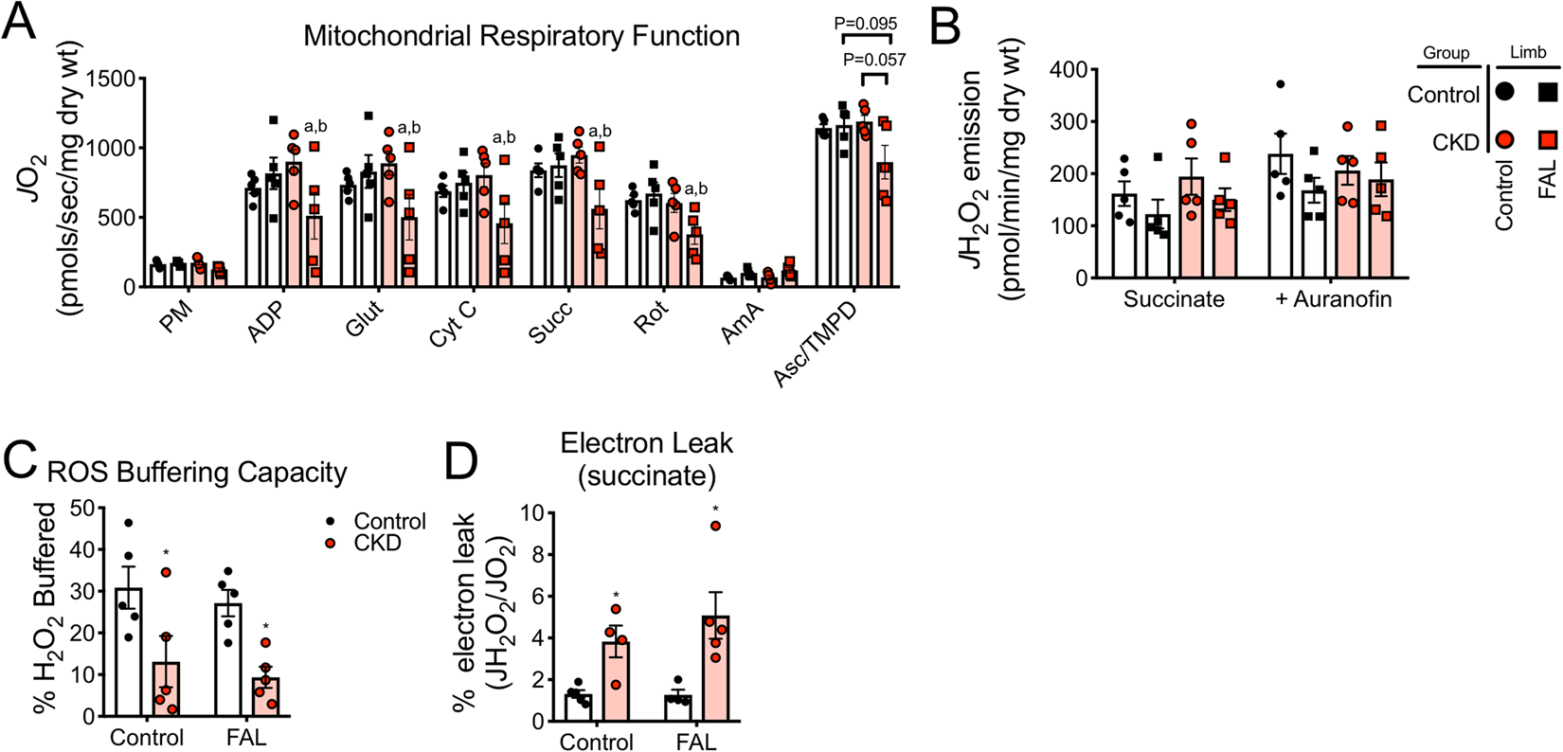
CKD mice experience severe skeletal muscle mitochondriopathy in the ischemic limb. Mitochondrial function was assessed *in situ* using permeabilized myofiber bundles prepared from the gastrocnemius muscles. (**A**) Mitochondrial respiratory function under numerous substrate conditions was significantly decreased in the ischemic muscle of CKD mice. (**B**) Mitochondrial hydrogen peroxide emission was slightly, but not statistically, elevated in CKD mice. (**C**) Calculated hydrogen peroxide buffering capacity (percent increase in H_2_O_2_ emission with auranofin) was lower in CKD mice. (**D**) Electron leak (*J*H_2_O_2_/*J*O_2_) supported by succinate (state 2) was also higher in CKD mice. *P < 0.05 vs. control mice. ^a^*P* < 0.05 vs. non-ischemic control (within group), ^b^*P* < 0.05 vs. control mice (between group, same limb) using ANOVA with Tukey’s post-hoc testing. N = 5/group. Error bars represent SEM. FAL = femoral artery ligation.

Previous work has linked skeletal muscle mitochondrial function with ischemic muscle injury in mice^27,55^. Similar to previous results in C57BL6/J mice^55^, mitochondrial respiratory function (*J*O_2_) was not different between control and ischemic limbs in mice receiving the casein-control diet (Fig. 5A). In contrast, CKD mice exhibited reduced (~50%) mitochondrial respiratory function in myofiber bundles prepared from ischemic limbs (Fig. 5A). Importantly, this impairment in mitochondrial function was observed across several substrate conditions. Next, mitochondrial hydrogen peroxide emission/production was assessed in myofiber bundles using Amplex UltraRed. Although not statistically significant, both control and ischemic myofibers bundles from CKD mice displayed a ~15–18% increase in hydrogen peroxide emission (Fig. 5B). During these experiments, antioxidant buffering capacity was assessed with the addition of auranofin (an inhibitor of thioredoxin reductase, a major buffering system in skeletal muscle^56^). ROS buffering capacity, calculated as the percentage increase in hydrogen peroxide emission upon the addition of auranofin, was substantially decreased in both control and ischemic limbs of CKD mice (Fig. 5C). Using parallel measurements of oxygen consumption, an estimation of electron leak was next calculated by dividing hydrogen peroxide emission rates by oxygen consumption under identical substrate conditions. Consistent with the modest increase in hydrogen peroxide emission, CKD mice had higher rates of electron leak in both control and ischemic limb muscle compared with control mice (Fig. 5D). These findings indicate that CKD exacerbates ischemic mitochondriopathy in skeletal muscle manifested by impaired oxidative phosphorylation and elevated oxidative stress.

### Serum from CKD mice induces myotube atrophy and oxidative stress

**Figure 6:**
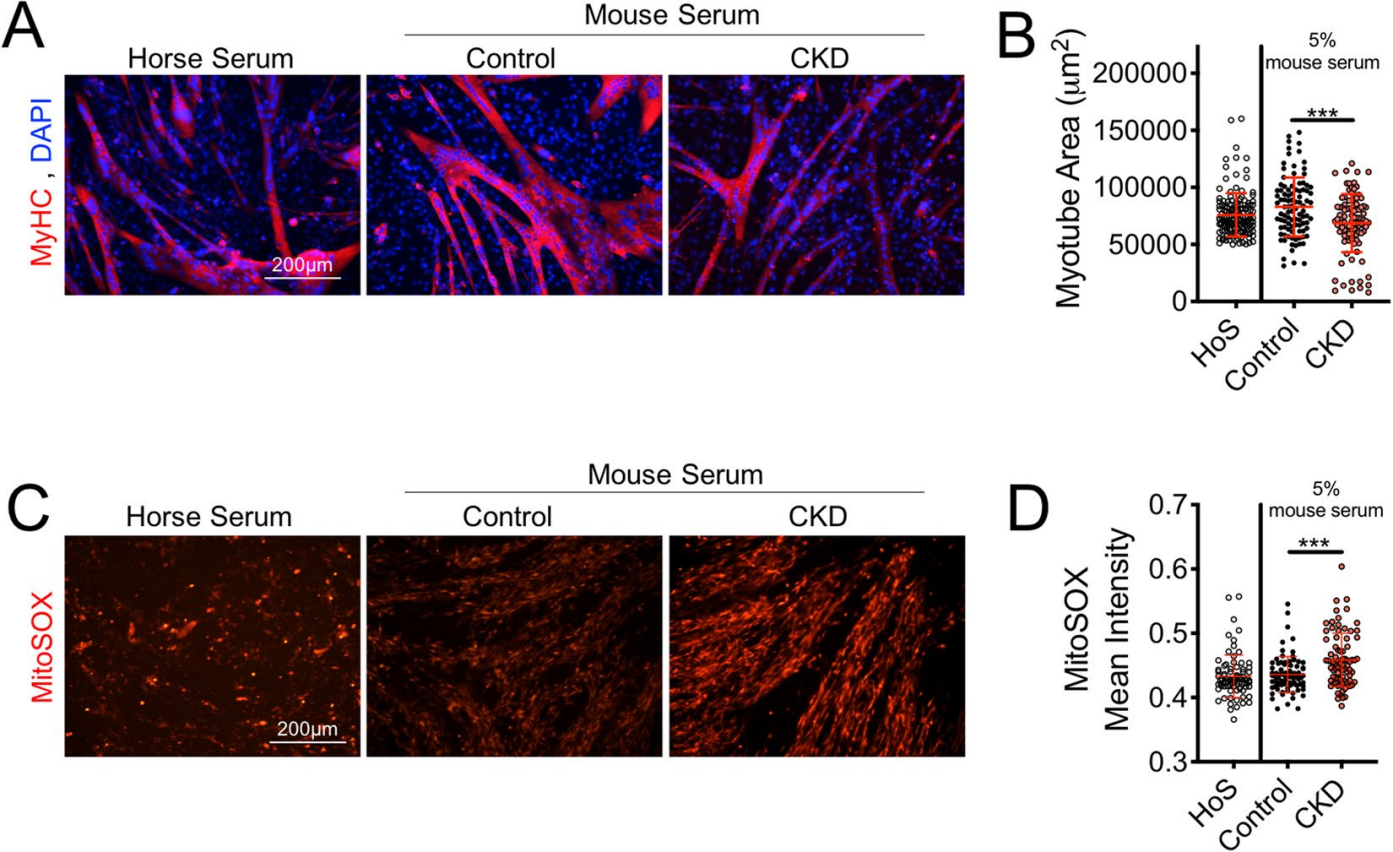
Serum from CKD mice causes myotube atrophy and elevates mitochondrial oxidative stress. Mature myotubes (C2C12) were exposed to serum collected from control and CKD mice at the time of sacrifice. Horse serum was used as the standard for myotube differentiation. (**A**,**B**) 24 h treatment with CKD mouse serum, but not control mouse serum, resulted in significant myotube atrophy. (**C**,**D**) 24 h treatment with CKD serum also resulted in increased mitoSOX staining intensity, an indicator of greater mitochondrial superoxide production. ***P < 0.001 vs. control ANOVA with Tukey’s post-hoc testing. Error bars represent SD. HoS = horse serum (standard differentiation medium).

A major function of the kidneys is to filter and remove waste products from the blood that are either ingested or produced endogenously through metabolism. CKD results in impaired kidney function that leads to the retention and accumulation of numerous solutes/metabolites, a condition described as uremia^57–59^. Some uremic metabolites, most prominently indoxyl sulfate, have recently received attention for negatively impacting muscle cell function^60,61^. To determine if uremia may play a role in the development of skeletal myopathy in CKD mice, a muscle cell (C2C12) culture model was employed. First, C2C12 myoblasts were differentiated into mature myotubes via serum withdrawal. Once mature myotubes were formed, differentiation medium was removed and replaced with DMEM supplemented with 5% serum collected from normal and CKD mice at sacrifice. Exposure of myotubes to CKD mouse serum for 24 h resulted in significant myotube atrophy, assessed by staining myotubes for sarcomeric myosin (MyHC; Fig. 6A,B). Because myotube atrophy may be a direct result of increased oxidative stress^62^, myotube ROS production was next assessed using a fluorgenic probe, MitoSOX, to measure mitochondrial-derived superoxide. Consistent with observations in myofibers bundles prepared from CKD mice, myotubes exposed to CKD mouse serum displayed increased MitoSOX fluorescence intensity (Fig. 6C,D), confirming increased levels of superoxide production.

**Figure 7:**
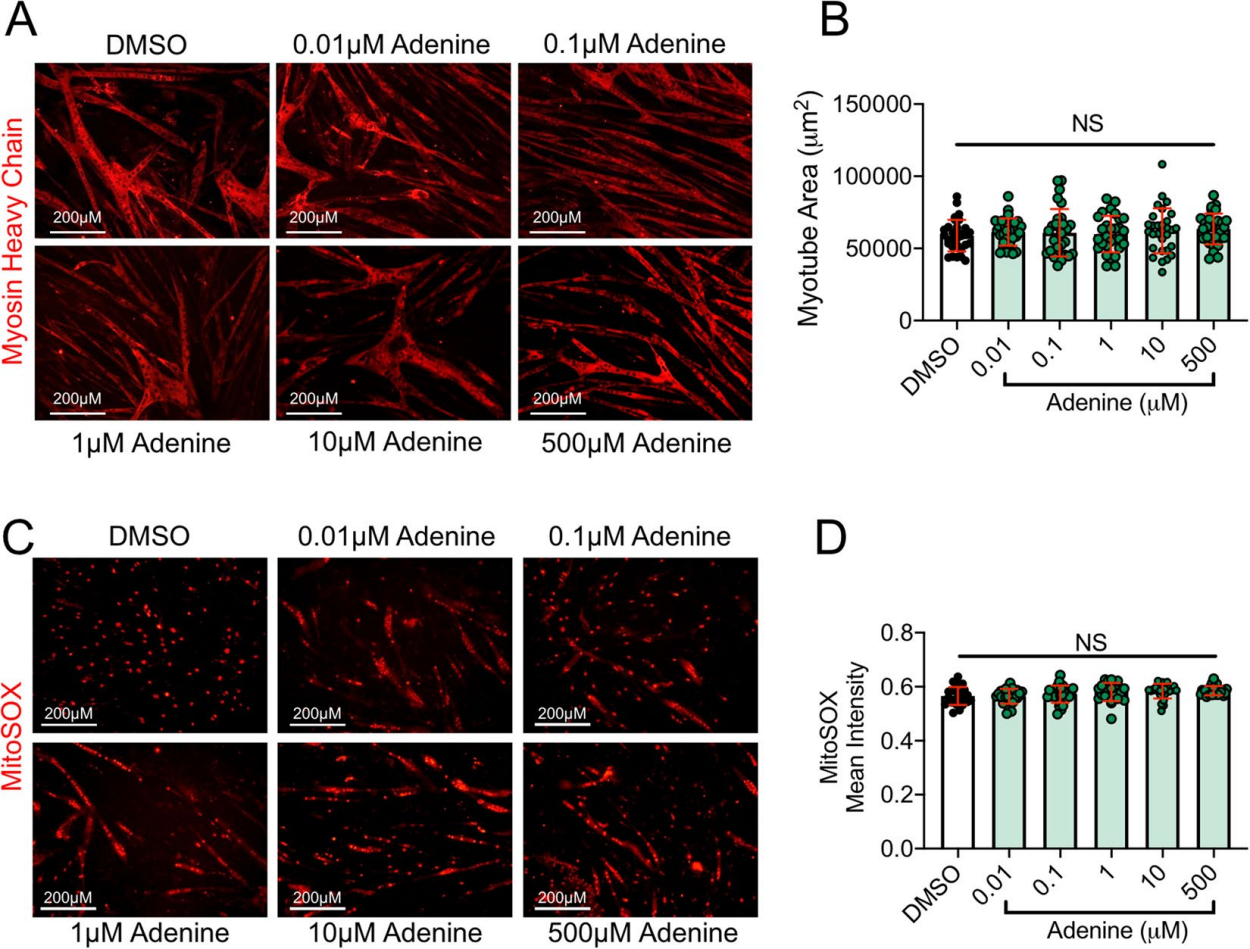
Adenine exposure does not induce myotube atrophy or mitochondrial oxidative stress. To examine if adenine alone results in toxicity of muscle cells, mature myotubes were exposed to increasing concentrations of adenine in culture for 24 hours. (**A**) Representative images of myotube size (myosin heavy chain area) and (**B**) quantification of myotube area following control (DMSO) and adenine treatment. (**C**) Representative images of mitoSOX staining in live myotubes and (**B**) quantification of mitoSOX intensity following control (DMSO) and adenine treatment. NS = not significant using ANOVA with Tukey’s post-hoc testing. Error bars represent SD.

To examine potential adenine toxicity in muscle cells, we next performed experiments by exposing myotubes to increasing concentrations of adenine. Adenine exposure (0.01 to 500 μM) for 24 hours did not result in myotube atrophy (Fig. 7A,B) or increase mitochondrial ROS production measured using mitoSOX staining in live myotubes (Fig. 7C,D). Together, this findings lend support to the hypothesis that uremic metabolites, but not adenine alone, are responsible for adverse muscle impacts caused by CKD serum^63^.

### Mitochondrial therapy decreases oxidative stress and rescues myotube atrophy with exposure to CKD mouse serum and hypoxia

**Figure 8:**
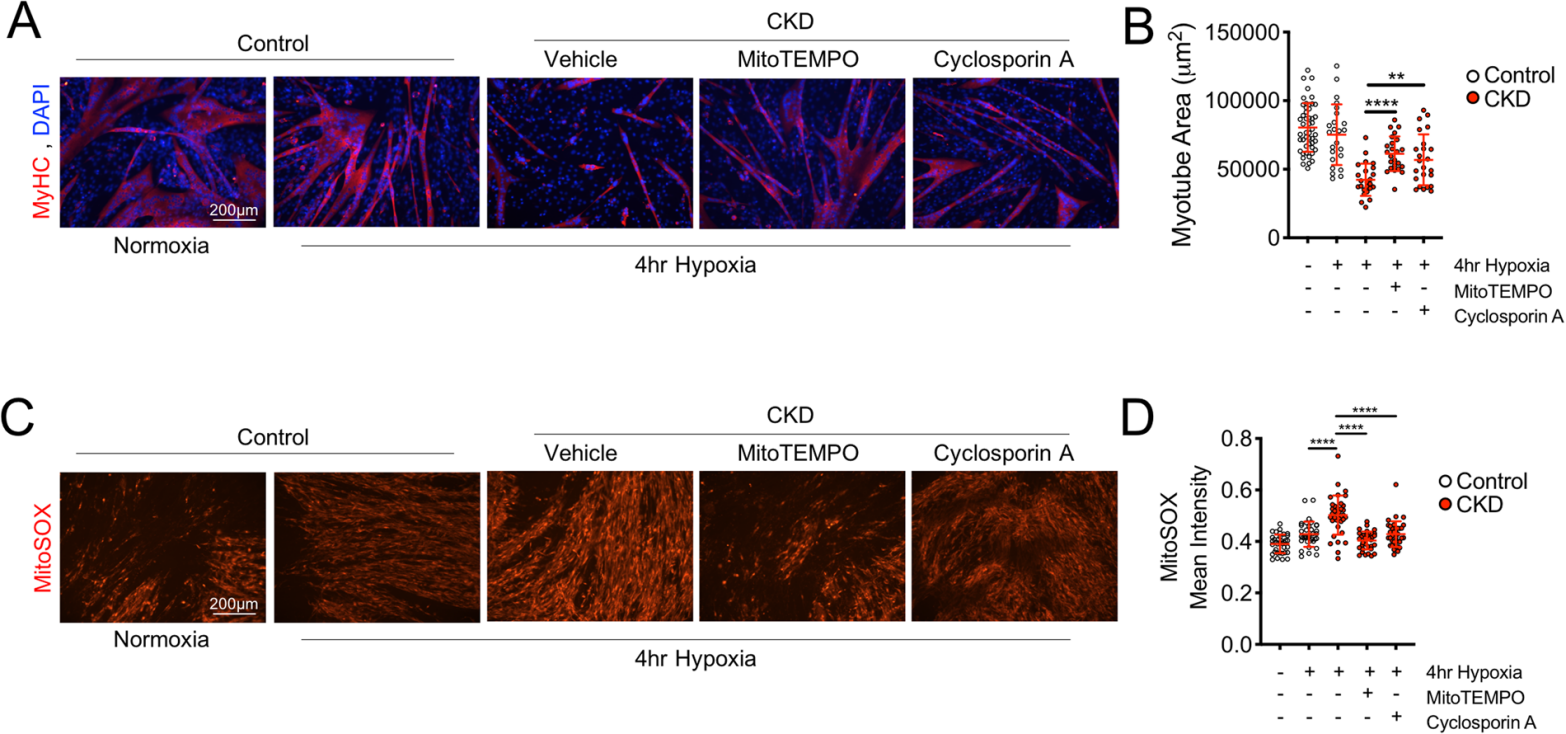
Mitochondrial therapies prevent myotube atrophy and reduce oxidative stress with CKD serum exposure and hypoxia treatment. Mature myotubes (C2C12) were treated with control mouse serum or CKD mouse serum with or without mitochondrial targeted therapy: mitoTEMPO (a mitochondrial antioxidant) or cyclosporin A (an inhibitor of mitochondrial permeability transition pore formation). (**A**,**B**) Treatment with CKD mouse serum and hypoxia resulted in substantial myotube atrophy which was largely prevented from co-treatment with mitoTEMPO (5 μM) or cyclosporin A (1 μM). (**C**,**D**) Treatment with CKD mouse serum and hypoxia elevated mitochondrial superoxide production compared with control serum, which was attenuated by co-treatment with either mitoTEMPO (5 μM) or cyclosporin A (1 μM). **P < 0.01, ***P < 0.001, ****P < 0.0001 using ANOVA with Tukey’s post-hoc testing. Error bars represent SD.

Based on the knowledge that mitochondrial oxidative stress can directly activate proteolytic and atrophy signaling pathways in skeletal muscle^62,64^, it was hypothesized that mitochondrial targeted therapies that reduce oxidative stress and/or prevent the formation of the mitochondrial permeability transition pore (mediator of cytochrome c release and subsequent cell death)^65^ might prevent the negative effects of CKD serum. To test this hypothesis, mature myotubes were treated with 5% serum from either control or CKD mice for 24 h, and then exposed to a 4 hr hypoxia treatment^27^ with or without treatment with mitoTEMPO (a mitochondrial targeted antioxidant) or cyclosporin A (which impairs the formation of the permeability transition pore). Supporting this hypothesis, both mitoTEMPO and cyclosporin A reduced myotube atrophy (Fig. 8A,B) and decreased mitochondrial superoxide production (Fig. 8C,D) with exposure to CKD serum and hypoxia. These findings highlight the therapeutic potential of mitochondrial targeted therapies for PAD patients with CKD and strongly suggests that future preclinical studies are necessary to further establish efficacy of these types of therapies.

## Discussion

There is an abundance of clinical and epidemiological data demonstrating that CKD is linked to worsened health outcomes in PAD patients^3,5–9,11,12,43^, however the biological mechanisms are not known. In this study, CLI patients with CKD displayed worsen ischemic myopathy characterized by reduced myofiber cross sectional area and a marked decreased in skeletal muscle mitochondrial respiratory function. Given that skeletal muscle health and function is the strongest predictor of PAD mortality^22,25,66^, this study aimed to examine the impact of CKD on the development of skeletal myopathy in mice subjected to a preclinical PAD model (femoral artery ligation). Using an inbred mouse strain that normally displays remarkable protection from ischemic injury^26,28,38,51–54,67^, induction of CKD abolished this genetic protection and resulted in the development of substantial ischemic muscle injury and impaired mitochondrial function. Importantly, muscle myopathy developed despite normal total capillary density in CKD mice. Additional muscle cell culture experiments suggest that serum metabolites that accumulate in CKD mice (uremia) result in elevated mitochondrial ROS production and myotube atrophy, both of which could be prevented using mitochondrial targeted therapies. Taken together, these findings implicate the development of severe skeletal myopathy as a potential mechanism for the increased amputation and mortality risk in PAD patients that have CKD, and establish mitochondrial energetics as a potential therapeutic avenue for improving PAD pathology in the presence of CKD.

There is a growing body of evidence that implicates skeletal myopathy as a mediator of PAD health outcomes. Several clinical studies have demonstrated that exercise capacity, physical activity, and walking performance are strong predictors of mortality in PAD patients^16,18,19,21,22,68^. Moreover, muscle strength^17,20^ and mitochondrial density/content^25^ have also been strongly linked to mortality of PAD patients. Interestingly, histological and biochemical evidence of skeletal myopathy has been previously reported in both PAD^69–76^ and CKD^77–81^ patients alone. Specifically, it has been shown that both PAD and CKD result in impairments of skeletal muscle mitochondrial function resulting in increased oxidative stress^26,27,70,71,73–76,78,81–92^. Despite this large body of literature, to our knowledge no studies have examined the development of ischemic skeletal myopathy in CKD. Herein, human CLI patients and mice with CKD displayed pronounced ischemic muscle injury and severe mitochondrial impairments compared with non-CKD controls. It should be noted that mice used in this study were C57BL/6 J mice, which have been previously shown to be remarkably resistant to ischemic muscle injury and mitochondrial impairments^28,38,52,53^. These findings suggest that the presence of CKD overrides the inherent genetic protections harbored by these inbred mice.

The few preclinical studies that performed femoral artery ligation on rodents with CKD have exclusively focused on vascular outcomes. Specifically, it was shown that nephrectomized rats subjected to femoral artery ligation displayed decreased limb perfusion recovery, lower muscle capillary density, and lower expression of VEGF and VEGFR-1 in ischemic muscles^93^. Another study in rats reported that CKD resulted in decreased expression of multiple genes involved in angiogenesis and vascular growth^94^. Interestingly, we observed normal perfusion recovery in the paw and tibialis anterior muscles of CKD mice, as well as normal capillary density. It is difficult to ascertain the reasons for the discrepancies in perfusion recovery/capillary density between our study and these previous studies because of the use of different species (mouse vs. rat), models of CKD (nephrectomy vs. adenine diet), and the timing of measurements following limb ischemia. Although limb perfusion is undoubtedly important in regards to PAD pathophysiology, it is notable that all cell- and gene-based angiogenic therapies tested in clinical trials with PAD patients have failed to demonstrate efficacy^95–100^. While the failure of vascular based trials is multifactorial, this observation suggests that targeting limb perfusion alone may not be the ideal therapeutic approach for PAD. Adjuvant therapies aimed to improve skeletal muscle health may prove beneficial, especially when considering its link to mortality and role as a paracrine signaling source for growing blood vessels^101^.

Using a muscle cell culture system, it was found that exposure of myotubes to serum taken from CKD mice resulted in elevated mitochondrial superoxide production and subsequent myotube atrophy. Impaired kidney function results in the accumulation of hundreds of water-soluble solutes and protein-bound metabolites that is termed uremia^57,58^. Importantly, many of the protein-bound uremic toxins are not sufficiently filtered by dialysis membranes^59,102^, and thus remain elevated in the blood despite treatment. To date, there is little known about the biological impact of most of these uremic toxins. Of the known uremic toxins, indoxyl sulfate is by far the most studied. Indoxyl sulfate is generated through endogenous tryptophan metabolism through the indolic pathway. It has been reported that indoxyl sulfate treatment results in impaired mitochondrial function in cultured muscle cells (C2C12), as well as muscle atrophy when delivered to mice^60,61^. Although uremic metabolites were not measured in serum obtained from CKD mice, the observed mitochondrial oxidative stress and atrophy are consistent with this previous work exposing muscle cells to indoxyl sulfate and recent discoveries that uremic metabolites disrupt mitochondrial energetics^63^. Moreover, elevated indoxyl sulfate in mice with CKD was linked to impaired endothelial progenitor cell driven neovascularization in mice subjected to hindlimb ischemia^103^, suggesting that uremic toxins may negatively impact both muscle and vascular tissues. The expected outcomes in both tissues would likely be additive in their negative impact on PAD pathophysiology. Future work is needed to further dissect the role of each uremic toxin on muscle and vascular cell biology.

In summary, the current study demonstrates that CKD results in exacerbation of ischemic muscle injury and the development of a severe mitochondriopathy in skeletal muscle. It was found that serum factors, likely the accumulation of uremic toxins, mediate these effects through alterations in mitochondrial oxidative stress. Future work is warranted to establish specific uremic toxins and develop novel treatment approaches aimed at the mitochondria to robustly improve ischemic outcomes in CKD.
